# C-952-05. Enhancing Frostbite Assessment Upon Admission Using Indocyanine Green (ICG) Perfusion Imaging: A Quality Improvement Initiative

**DOI:** 10.1093/jbcr/irag033.121

**Published:** 2026-04-06

**Authors:** Stephanie L Steiner, Anjay Khandelwal, Richard B Lou

**Affiliations:** Akron Children's Hospital Adult and Pediatric Burn Institute, Akron, OH; Akron Children's Hospital, Akron, OH; Akron Children's Hospital, Akron, OH

## Abstract

**Introduction:**

During the 2024 frostbite season, our regional Burn Institute cared for both adult and pediatric patients presenting with varying degrees of frostbite, most commonly involving the hands and feet. While some patients may arrive within 24 hours of initial rewarming after prolonged cold exposure, others are often complicated by delayed access to definitive care or delays in transfer to the Burn Institute. Historically, frostbite assessment and intervention were limited by the reliance on delayed nuclear imaging and unstandardized clinical practices. These challenges often resulted in inconsistent care and missed windows for thrombolytic therapy.

**Methods:**

In Fall 2024, we launched a multidisciplinary quality improvement (QI) initiative to improve early time sensitive frostbite management. The initiative centered on the implementation of bedside indocyanine green (ICG) perfusion scan using a fluorescent imaging system. A standardized Frostbite Guideline was expanded to include early ICG imaging upon presentation to our Burn Institute. A detailed Standard Work Instruction (SWI) was created to guide bedside use of the fluorescent imaging system, including dosing protocols, scan sensitivity settings, and documentation. Clinical teams incorporated ICG findings into decision-making around thrombolytic therapy and anticoagulation therapies to arrive at timely treatment decisions.

**Results:**

Preliminary data from the 2024 season, though still under review, suggest significant improvements in early management. All eligible patients received ICG scans within just a few hours of presentation to the Burn Institute. Time to definitive treatment decisions—including thrombolytic therapy, anticoagulation initiation, and surgical planning—was reduced by more than 50% compared to prior frostbite seasons. Providers reported increased confidence in perfusion assessments, and earlier intervention was associated with improved tissue salvage in initial cases based on subjective surgical assessment. Nuclear scans, previously relied on for management decisions, were used as adjunct confirmation rather than primary guidance.

**Conclusions:**

Early bedside ICG perfusion imaging appears to be a feasible, rapid, and effective tool for standardizing frostbite assessment and guiding timely intervention. The structured Frostbite Guideline and SWI, helped reduce variability in care and allowed clinical teams to act within the narrow thrombolytic window, potentially improving outcomes.

**Applicability of Research to Practice:**

This QI project highlights a novel, replicable approach to frostbite care that may inform future clinical guidelines and multicenter research. The integration of ICG imaging offers a practical alternative to nuclear scanning, particularly in centers where imaging delays limit timely treatment. Further prospective studies are warranted to validate outcome improvements and support broader adoption of this technique.

**Funding for the study:**

N/A.

